# The global prevalence of screen-based disordered eating and associated risk factors among high school students: systematic review, meta-analysis, and meta-regression

**DOI:** 10.1186/s40337-023-00849-1

**Published:** 2023-08-03

**Authors:** Hadeel A. Ghazzawi, Lana S. Nimer, Dima H. Sweidan, Omar A. Alhaj, Duha Abulawi, Adam T. Amawi, Michael P. Levine, Haitham Jahrami

**Affiliations:** 1 Department Nutrition and Food Technology, School of Agriculture, The University of Jordan, P.O. Box 11942, Amman, Jordan; 2 Department of Nutrition, Faculty of Pharmacy and Medical Sciences, University of Petra, Amman, Jordan; 3 Department of Physical and Health Education, Faculty of Educational Sciences, Al-Ahliyya Amman University, As-Salt, 19328 Jordan; 4 Department of Psychology, Kenyon College, Gambier, OH 43022 USA; 5 Goverment Hospitals, Manama, Bahrain; 6 Department of Psychiatry, College of Medicine and Medical Sciences, Arabian Gulf University, Manama, Bahrain

**Keywords:** High school age, Students, Eating disorders, Disordered eating, Screening instruments

## Abstract

**Objective:**

Estimate the prevalence, and associated risk factors, of high school students who are considered at risk for an eating disorder based on screening measures.

**Methods:**

An electronic search of nine databases was completed from their inception until 1st September 2022. A random-effects meta-analysis was conducted, and confounder (moderator) analyses and meta-regressions examined whether the overall prevalence estimate for of screen-based disordered eating (SBDE) was moderated by student age, BMI, or gender, as well as culture and type of SBDE assessment.

**Results:**

The mean estimate of the prevalence of SBDE among high school students (*K* = 42 (66 datapoints), *N* = 56282] in the sample of 25 countries was 13% ([95% CI] = 10.0–16.8%, *I*^2^ = 99.0%, Cochran's Q *p* = 0.001). This effect was not moderated by features of the samples such as gender, BMI, or age. Among cultures, non-Western countries had a higher prevalence of SBDE prevalence than Western countries, but the difference was not significant. There was considerable variability in the prevalence estimates as a function of the assessment measure, but no meaningful pattern emerged.

**Conclusion:**

The estimated figure of 1 in 8 high school students with SBDE—unmoderated by gender and BMI—stands out as a problem in need of attention from public health officials, psychologists, psychiatrists, pediatricians, parents, and educators. There is a great need for innovative, integrated policy and program development all along the spectrum of health promotion and universal, selective, and indicated prevention. Further research is also needed to validate and refine this estimate by (a) conducting basic research on the accuracy of eating disorder screening measurements in samples ages 14 through 17; (b) examining representative samples in more countries in general and Latin American countries in particular; (c) clarifying the relationships between SBDE and age throughout the different phases of late childhood, adolescence, and emerging adulthood; and (d) investigating whether there are meaningful forms of disordered eating and whether these are associated with variables such as gender, ethnicity, and BMI.

## Plain Language summary

We searched nine databases to identify studies of high school students that yielded an estimate of disordered eating based on screening measures such as the Eating Attitudes Test. Forty-two 42 studies (*N* = 56282 students) from 25 countries met the selection criteria. A random effects meta-analysis indicated that across those countries the best estimate of the prevalence of screen-based disordered eating is 13%. This estimate was not significantly moderated by BMI, gender, age, and whether the country was Western or non-Western. There was considerable variability in the prevalence estimates as a function of the assessment measure, but no meaningful pattern emerged. The estimated figure of 1 in 8 high school students with disordered eating is a problem deserving of attention from public health officials, psychologists, psychiatrists, pediatricians, parents, educators, and leaders committed to prevention and early identification of eating disorders and referral for treatment. Further research in many more countries is also needed to validate this estimate and to explore its relationship with development throughout adolescence and with variables that can help us to refine prevention and effective early identification and treatment of eating disorders.

## Introduction

Eating disorders (EDs) are serious, all too often chronic, mental illnesses that usually begin in adolescence or emerging adulthood [1]. A recent review by Silén and Keski-Rahkonen indicates that “overall, 5.5–17.9% of young women and 0.6–2.4% of young men have experienced a DSM-5 eating disorder by early adulthood” [2]. Thus, approximately 30 million people globally suffer from EDs, and these disorders are frequently misdiagnosed and under- or ineffectively treated [3]. There is general agreement that a variable and complicated combination of biological, psychological, social, and cultural factors increases the risk of an ED [4].

Clinically recognized eating disorders such as anorexia nervosa (AN) and bulimia nervosa (BN) represent, at least in part, the extreme ends of a set of interlocking continua of characteristics, including negative body image, dietary restraint, and prominence of weight and shape in defining and evaluating the self. This continuity assumption is seen most prominently in the widespread use of the constructs of “disordered eating” (DE) and “screening for at-risk people”. Consider two broad categories of people that correspond to two broad types of measures used in the voluminous research on disordered eating: (1) people who demonstrate DE based on their high scores on focused measures of, for example, specific ED symptoms or aspects of “ED psychopathology”; and (2) those designated as “at-risk” based on their above-threshold scores on a valid screening instrument such as the Eating Attitudes Test (EAT) or the questionnaire version of Eating Disorders Examination (EDE-Q). In this study, as in our previous research (7), we focus on people in the latter category and consider them to “have” or exhibit *screen-based disordered eating* (SBDE).

People in either category would not currently meet accepted DSM-5 or ICD-11 criteria for an ED, nor have they previously had a clinically recognized ED and thus are at risk for relapse. Rather, these two groups of research participants—who almost certainly are representative of people in general populations—are understood to be at risk for an ED because of their current level of maladaptive beliefs, emotional responses, and behaviors. For example, longitudinal risk factor research consistently shows that negative body image and DE are the best predictors of the development of full-blown EDs, at least in adolescent girls and adult women [4–6].

Thus, one way of understanding DE is as an “at risk” status. Specifically, components of what many researchers consider DE, based on narrower definitions (category 1 above) constitute a large proportion of the items on the measures used to screen people to determine, relatively quickly and at low cost, who is “at risk” for actually having an ED (category 2 above) upon closer examination, using a structured diagnostic interview. In the present study we focus on SBDE because this construct is broader and more syndrome-like than the specific symptom- or psychopathology measures. The construct of SBDE also is more representative of Levine and Smolak’s (8,9) initial attempts at defining definition developed Using a prototypical approach such as that seen in many DSM-5 diagnostic algorithms, these researchers define DE as (1) “subclinical” but unhealthy, maladaptive, and misery-inducing levels of negative body image, weight and shape concerns, and dietary restrictions and/or binge eating; plus (2) at least two of the following: (a) individual eating disorder symptoms such as self-induced vomiting after eating; (b) abuse of laxatives, diuretics, diet pills, and exercise; (c) unrealistic beauty standards, including an idealization of thinness; (d) irrational and maladaptive beliefs about body fat and fat people, often coupled with a high drive for thinness; (e) relatively high levels of negative affect that the person finds difficult to tolerate and manage; and (f) harsh self-surveillance and self-criticism, often in transaction with low and unstable self-esteem.

Of the various screening instruments, the most widely used with the strongest psychometric properties for adults are The Eating Disorder Inventory (EDI), the Eating Attitudes Test (EAT), Eating Disorder Examination Questionnaire (EDE-Q), and the Sick, Control, One, Fat, Food (SCOFF). We acknowledge that their use as self-report tools for screening high school students to determine who is at risk for eating disorders is beset with tantalizing instances of support along with troubling inconsistencies and many unknowns [7–10]. Thus, we see this meta-analysis as a first and broad step in developing a reasonable and estimate, based on the current literature, of the prevalence of one general form of disordered eating [11].

Noting the lack of a previously agreed upon definition of DE and thus the lack of research data about its point prevalence, Levine and Smolak [11] estimated 15–20%, based on studies of either the prevalence of individual ED symptoms or the percentage of people scoring above cut-offs on measures such as the Eating Attitudes Test (EAT). Our recent meta-analysis of 89 studies of SBDE in university students, conducted in 40 countries and territories (*K* = 105, *N* = 149,629), yielded a prevalence of 19.7% [12]. However, there was significant heterogeneity in the point prevalence of SBDE as a function of the type of measure. This is likely due to several factors, including variation in the operational definitions of and specific items for assessing DE, differences in the study populations, and limitations of the assessment tools themselves. For example, the SCOFF has only 5 items and, unlike the much longer EDE-Q, does not address the centrality of weight/shape for self-definition; in turn, the EDE-Q, unlike the EAT-26 does not specifically assess binge eating. This conceptual variability and our meta-analytic findings highlight Levine and Smolak’s contention that, while useful and meaningful, DE is a complex and multifaceted construct (or family of constructs) that encompasses a range of behaviors related to food, weight, and body image.

Although they are rare, EDs do indeed occur in late childhood and preadolescence, and these certainly deserve the attention of researchers, mental health professionals, and medical professionals [13, 14]. Nevertheless, adolescence, broadly defined as ages 11 through 19, is a period of elevated risk for EDs, culminating in the modal ages of onset, roughly 17 through 22, that is, late adolescence and emerging adulthood [15]. In the context of most cultures, the biopsychosocial changes captured in the phrase “adolescent development” unfold in the context of a set of generally recognized developmental tasks (i.e., needs and sociocultural expectations). These include defining and deepening friendship networks; accepting and appreciating physical development; constructing a stable but flexible identity, including gender roles; and establishing autonomy from, while redefining attachment to, one’s family [14, 16]. A wide variety of normative (e.g., weight bias, teasing based on physical appearance, sexual objectification, and the cultural glorification of thinness), somewhat normative (e.g., parental divorce, increased pressure in sports, academics, or dance), and non-normative factors and stressors (e.g., sexual trauma, emergence of an illness such as diabetes) can make negotiation of adolescence and its developmental tasks very difficult. These factors, particularly in combination, clearly set the stage for the emergence of DE, EDs, and related conditions (e.g., depression) during adolescence [5, 11].

We know that late adolescence and emerging adulthood are periods of high risk for EDs; as noted previously, roughly 1 in 5 people in that age range worldwide report DE, based on scores greater than the established “risk” cut-off scores on screening measures [11]. We know that some, if not a great many, of these people *and* of those whose EDs emerged during that developmental phase were showing ED symptoms and signs during middle adolescence. We also know that DE is a public health concern in and of itself because of its links with depression, anxiety, binge drinking, cigarette smoking, the extremes of physical in/activity, and self-harm [17–20]. Nevertheless, it has been difficult to determine the percentage of those of high school age, defined as ages 14 through 18, who report SBDE. For example, as shown in Table 1, estimates from studies conducted in the USA, using validated screening instruments in samples with a mean age of 15 or 16, have varied from 14% [21, 22] to 35% [23] to 56% [24]. Among the studies of high school students with those mean ages conducted in other countries, estimates have varied from 1% in Italy [25] to 67% in Brazil [26].

**Table 1:** Characteristics of the studies involved in the systematic review and meta-analysis about the prevalence of disordered eating in high school students

S. No.	References	Country	COVID-19	Design	Sample	Measure	Population characteristics	Prevalence (%)	Quality Score
1	Al-sheyab et al. [27]	Jordan	No	Cross-sectional	738	EAT- 26	Female^%^ = 55.3%, Age_Mean_ = 15 years, BMI_Mean_ = 21 kg/m^2^	24	8
2	Bould et al. [28]	UK	No	Cohort study	1769	DAWBA	Female^%^ = 100%, Age_Mean_ = 16 years, BMI_Mean_ = 21 kg/m^2^	41	8
3	Canals et al. [29]	Spain	No	Cross-sectional	515	EAT-40	Female^%^ = 43.7%, Age_Mean_ = 16 years, BMI_Mean_ = 21 kg/m^2^	10	8
4	Caradas et al. [30]	Africa	No	Cross-sectional	228	EAT-26	Female^%^ = 100%, Age_Mean_ = 16 years, BMI_Mean_ = 23 kg/m^2^	18	5
5	Cheah et al. [31]	Malaysia	No	Cross-sectional	329	EAT-26	Female^%^ = 59%, Age_Mean_ = 16 years, BMI_Mean_ = 21 kg/m^2^	19	5
6	Cotrufo et al. [32]	Italy	No	Cross-sectional	356	EDI 2	Female^%^ = 100%, Age_Mean_ = 16 years, BMI_Mean_ = 21 kg/m^2^	30	5
7	de Souza Ferreira and da Veiga [26]	Brazil	No	Cross-sectional	561	EDE-Q	Female^%^ = 62.9%, Age_Mean_ = 16 years, BMI_Mean_ = 21 kg/m^2^	67	8
8	Devaud et al. [33]	Switzerland	No	Cross-sectional	2501	PEC, WIC	Female^%^ = 43.3%, Age_Mean_ = 16 years, BMI_Mean_ = 21 kg/m^2^	3	8
9	Eapen et al. [34]	UAE	No	Cross-sectional	495	EAT-40	Female^%^ = 100%, Age_Mean_ = 16 years, BMI_Mean_ = 21 kg/m^2^	23	8
10	Fatima and Ahmad [35]	Saudi Arabia	No	Cross-sectional	314	EAT- 26	Female^%^ = 100%, Age_Mean_ = 17 years, BMI_Mean_ = 21 kg/m^2^	25	5
11	Hautala et al. [36]	Finland	No	Cross-sectional	1036	SCOFF	Female^%^ = 54%, Age_Mean_ = 16 years, BMI_Mean_ = 21 kg/m^2^	20	8
12	Jones et al. [37]	Canada	No	Cross-sectional	1739	EAT-26, DSED	Female^%^ = 100%, Age_Mean_ = 15 years, BMI_Mean_ = 21 kg/m^2^	14	8
13	Koushiou et al. [38]	Greece	No	Cross-sectional	741	EDDS	Female^%^ = 63%, Age_Mean_ = 15 years, BMI_Mean_ = 21 kg/m^2^	9	8
14	Le Grange et al. [39]	South Africa	No	Cross-sectional	813	EAT-26	Female^%^ = 58%, Age_Mean_ = 17 years, BMI_Mean_ = 21 kg/m^2^	18	8
15	Makdad et al. [17]	Morocco	No	Cross-sectional	367	EAT- 26	Female^%^ = 51.5%, Age_Mean_ = 16 years, BMI_Mean_ = 20 kg/m^2^	10	5
16	Maor et al. [40]	Israel	No	Cross-sectional	245	EAT-26	Female^%^ = 51%, Age_Mean_ = 16 years, BMI_Mean_ = 21 kg/m^2^	13	5
17	Martinsen et al. [41]	Norway	No	Case–control	606	EDI-2	Female^%^ = 35.8%, Age_Mean_ = 16 years, BMI_Mean_ = 21 kg/m^2^	24	8
18	Miller et al. [21]	USA	No	Cross-sectional	1302	EAT40	Female^%^ = 62.75%, Age_Mean_ = 15 years, BMI_Mean_ = 22 kg/m^2^	14	8
19	Miotto et al. [42]	Italy	No	Cross-sectional	903	EAT, BITE, BAT	Female^%^ = 69.21%, Age_Mean_ = 17 years, BMI_Mean_ = 21 kg/m^2^	12	8
20	Mohiti et al. [43]	Iran	No	Cross-sectional	359	EAT-26	Female^%^ = 100%, Age_Mean_ = 16 years, BMI_Mean_ = 22 kg/m^2^	22	5
21	Mond et al. [44]	Australia	No	Cross-sectional	1664	EDE-Q	Female^%^ = 68%, Age_Mean_ = 15 years, BMI_Mean_ = 21 kg/m^2^	23	8
23	Nichols et al. [45]	USA	No	Cross-sectional	170	EDE-Q	Female^%^ = 100%, Age_Mean_ = 16 years, BMI_Mean_ = 22 kg/m^2^	18	5
22	Nichols et al. [46]	USA	No	Cross-sectional	423	EDE-Q	Female^%^ = 100%, Age_Mean_ = 16 years, BMI_Mean_ = 22 kg/m^2^	20	8
24	Pastore et al. [47]	USA	No	Cross-sectional	1001	EAT-26	Female^%^ = 55%, Age_Mean_ = 16 years, BMI_Mean_ = 21 kg/m^2^	12	8
26	Patton et al. [48]	Australia	No	Cohort study	853	BET	Female^%^ = 100%, Age_Mean_ = 16 years, BMI_Mean_ = 21 kg/m^2^	7	8
27	Preti et al. [25]	Italy	No	Cross-sectional	828	EAT, BITE, BAT	Female^%^ = 64.61%, Age_Mean_ = 17 years, BMI_Mean_ = 21 kg/m^2^	9	8
28	Preti et al. [25]	Italy	No	Cross-sectional	817	EAT,BITE	Female^%^ = 65.5%, Age_Mean_ = 17 years, BMI_Mean_ = 21 kg/m^2^	1	8
29	Pustivšek et al. [49]	Slovenia	No	Cross-sectional	583	SCOFF	Female^%^ = 46.83%, Age_Mean_ = 16 years, BMI_Mean_ = 21 kg/m^2^	39	8
30	Rathner and Messner [50]	Italy	No	Cohort study	517	EAT-40, EAT-26	Female^%^ = 100%, Age_Mean_ = 15 years, BMI_Mean_ = 21 kg/m^2^	3	8
31	Robinson et al. [51]	Multi	No	Cohort study	1509	DAWBA	Female^%^ = 50%, Age_Mean_ = 15 years, BMI_Mean_ = 21 kg/m^2^	20	8
32	Sancho et al. [52]	Spain	No	Cohort study	1336	ChEAT, DICA-C, DICA-A, BITE	Female^%^ = 51.42%, Age_Mean_ = 11 years, BMI_Mean_ = 21 kg/m^2^	13	8
33	Stachowitz et al. [53]	USA	No	Cross-sectional	65	EDI-3	Female^%^ = 100%, Age_Mean_ = 15 years, BMI_Mean_ = 21 kg/m^2^	11	6
34	Štefanová et al. [54]	Slovakia	No	Cross-sectional	780	SCOFF	Female^%^ = 44%, Age_Mean_ = 14 years, BMI_Mean_ = 21 kg/m^2^	27	8
35	Szabo and Hollands [55]	Africa	No	Cross-sectional	213	EAT- 26	Female^%^ = 100%, Age_Mean_ = 15 years, BMI_Mean_ = 19 kg/m^2^	22	6
36	Tao [56]	China	No	Cohort study	1199	EAT-26	Female^%^ = 63.9%, Age_Mean_ = 19 years, BMI_Mean_ = 21 kg/m^2^	10	8
37	Thein-Nissenbaum et al. [23]	USA	No	Cohort study	311	EDE-Q	Female^%^ = 100%, Age_Mean_ = 15 years, BMI_Mean_ = 21 kg/m^2^	35	6
38	Thein-Nissenbaum et al. [24]	USA	No	Cohort study	43	EDE-Q	Female^%^ = 100%, Age_Mean_ = 16 years, BMI_Mean_ = 22 kg/m^2^	56	6
39	Thomas et al. [57]	USA	No	Cross-sectional	63	EDI	Female^%^ = 100%, Age_Mean_ = 15 years, BMI_Mean_ = 18 kg/m^2^	30	6
40	Torstveit et al. [58]	Norway	No	Cross-sectional	2451	EDI-2	Female^%^ = 51.16%, Age_Mean_ = 16 years, BMI_Mean_ = 22 kg/m^2^	55	8
41	Tseng et al. [59]	Taiwan	No	Cross-sectional	1794	EAT-26, BITE	Female^%^ = 100%, Age_Mean_ = 16 years, BMI_Mean_ = 20 kg/m^2^	4	8
42	Vega Alonso et al. [60]	Spain	No	Cohort study	2480	EAT-40	Female^%^ = 50.8%, Age_Mean_ = 16 years, BMI_Mean_ = 21 kg/m^2^	8	8

*FEDS* Feeding and eating disorders. Quality score was computed based on Newcastle-Ottawa quality assessment scale total score for cross-sectional studies

*EAT-26* Eating attitudes test-26, *EAT-40* Eating attitudes test-40, *SCOFF* Sick, control, one stone, fat, food, *EDE-Q* Eating disorder examination- questionnaire, *BEDS-7* Binge eating disorder screener-7, *ORTO-15*= ORTO-15, *QEDD* Questionnaire for eating disorder diagnoses, *EDDS* The eating disorder diagnostic scale, *SD* Self-developed, *WCS* The weight concern scale, *DEBQ* Dutch eating behavior questionnaire, *EDI* Eating disorder inventory-I/II, *ORTO-11* ORTO-11, *ANIS* Anorexia nervosa inventory for self-rating

To address this confusing state of affairs and to extend our previous meta-analytic reviews of SBDE in older adolescents and emerging adults [12, 61], we conducted a meta-analysis of the global prevalence of SBDE in high school students. To the best of our knowledge, based on searches of the literature or other registration platforms, this is the first such meta-analysis of DE and potential moderators/confounders in this population. As noted above, the prevalence estimate for each study is based on a pre-defined cut-off score from the particular validated screening device(s)—that is, a continuous measure of ED risk such as the EAT-26 and SCOFF (see Table 1)—used in the study. In the present meta-analysis of SBDE, the moderators/confounders examined were gender (male or female), BMI, age, and Western vs. non-Western countries. Based on previous risk factor research and our meta-analysis of SBDE in university undergraduates [12], it was predicted that the prevalence of SBDE would be greater in samples with (a) a greater ratio of females to males [62–64]; (b) higher mean BMI scores [65, 66]; and (c) a mean age in high school that is closer to the modal age of ED onset (ages 18–24; [15]. We also examined year of publication and type of measure, although, as was the case for the limited concept of “Westernization” [6] we did not have specific hypotheses in regard to these variables.

## Materials and methods

This systematic review, registered in PROSPERO (CRD42022353763), was conducted using the Preferred Reporting Items for Systematic Reviews and Meta-Analyses (PRISMA2020; [67] and the Meta-analysis of Observational Studies in Epidemiology (MOOSE) procedure [68].

### Search strategy

In August 2022, two authors (HJ and DS) used nine databases to perform the electronic literature search. The following keywords and lists were included in the full-text search: List A: School student [OR] adolescent [OR] adolescence [OR] high school student [AND] List B: eating behavior/behavior [OR] eating disorder [OR] feeding disorder [OR] eating problem [OR] eating symptom [OR] eating attitude. Use of the asterisk symbol assures that the search considers both single noun forms and a phrase's words in reverse order. For instance, looking for “eating disorder*” covers both “disordered eating” in addition to “eating disorders”. Electronic searches were performed in PubMed/MEDLINE, PsycINFO, Cochrane Library, Embase, Scopus, CINAHL, and Web of Science. Our initial goal here was to identify potentially relevant publications in reputable journals, defined as academic or scientific publications that are peer-reviewed and indexed in a scientific database such as PsychINFO.

To ensure that all relevant publications were included, the reference lists of articles selected from those journals were examined. In addition, because meta-analyses might provide inflated effect size estimations if they exclude grey literature, while examining the reference sections of relevant articles we looked for organizational reports, research published outside of reputable journals, and unpublished studies. Grey literature refers to information that is not published through traditional commercial or academic channels, such as conference proceedings, working papers, government reports, and other non-peer-reviewed publications. In addition to examining reference sections in journal articles, a literature search was performed in OpenGrey and GreyNet databases.

After excluding duplicate studies, the article titles, abstracts, and manuscripts (full text) were further screened by two team members (DS AND LN), and then the initial group of studies resulting from this step was independently assessed by three team members (DS, LN, and HJ). Four team members (DS, LN, HJ and OA) individually extracted the preliminary data and quality evaluation. Any differences of opinion regarding the suitability of reviewing this study based on inclusion or exclusion criteria were revised by dialogue with the leading reviewers/expert clinicians (HG, OA, HJ), then by unanimous agreement of the review study group.

### Eligibility criteria

Studies were accepted for inclusion in the meta-analysis if they met all the following criteria: (1) published in an English- or Arabic-language journal; (2) either the whole sample or a distinct group consisted of high school students; and (3) in order to determine who is at risk for an ED, participants completed at least one of the valid screening tests listed in Table 1. If, after contacting the authors, we were still unable to determine whether a study met all three of those criteria, that study was excluded from the analyses.

### Procedure

For screening and coding of the 42 studies (contributing 66 data points, due to multiple screening tools or multiple data collection times) ultimately selected for meta-analytic review, ASReview was used. This tool (available at https://asreview.nl/) is a free online resource that incorporates digital technologies and uses machine learning and artificial intelligence [69]. ASReview is designed to be user-friendly and can be easily integrated with existing literature review workflows. The tool operates in four main stages to automate the systematic reviewing of large volumes of scientific literature: (a) importing the dataset of articles to be screened; (b) screening articles using machine learning algorithms; (c) reviewing and resolving any conflicts or uncertainties in the screening process; and (d) exporting the final set of relevant articles.

In order to standardize data description and identify potential moderators of effect size, two members (DS and LN) of the research team collected data for the following variables: names of the authors; year of publication; the nation where the data were gathered; sample size; average age (in years); proportion of the sample that self-identified as female; and the measure used to assess SBDE. This meta-analysis included samples from 25 countries. They were further categorized into Western and non-Western countries based on the United Nations regional groups of member states.

Assessment of interrater accuracy in screening articles according to the inclusion and exclusion criteria resulted in an agreement rate of 97% between the two reviewers (DS and LN). After discussion and dialogue with a third expert reviewer/meta-analyst (HJ), the agreement rate increased to 100%. This indicates that the third reviewer was able to help resolve the relatively few discrepancies or disagreements that occurred between the initial reviewers, resulting in a consensus on all of the documents or data that were evaluated.

### Assessment of quality and risk of bias

The quality of the studies accepted for meta-analytic review was independently evaluated by two members of the research team (HG and HJ) using the Newcastle-Ottawa Scale (NOS; [70]. Assessment of interrater accuracy in quality assessment resulted in an agreement rate of 100% between the two reviewers (HG and HJ). Three items make up the NOS checklist: participant selection (sampling), comparability of cases and controls, and results and statistics. Each item receives a rating of 1 to 3 (or 4) stars, so the maximum score for each study is either 9 (cross-sectional and cohort studies) or 10 stars (randomized controlled trials and case-control studies). A study with 8 or more stars has good quality and low risk of bias, 5–7 stars indicates moderate quality and moderate risk of bias, and 0–4 stars indicates low quality and high risk of bias. A traffic light graphic was generated to represent the bias risk in each domain (participants’’ selection, comparability and analytics, and outcome measurement) and the total risk.

### Data analysis and data visualization

All data were analyzed using the R software for statistical computing [the R foundation for statistical computing, p. 9] and ‘metafor’ [71]. There are two main approaches to calculating effect sizes in a meta-analysis [72]: random-effects models and fixed-effects models. In a fixed-effects (aka common effect) model, it is assumed that all studies entered into the meta-analysis share a common effect size, so any differences in effect sizes between studies are due to chance or sampling error. This model is appropriate when the studies in the meta-analysis are very similar (homogenous) in terms of their design, participants, and intervention or exposure.

In contrast, a random-effects model assumes that there is variability in the true effect sizes across studies, beyond what can be explained by chance or sampling error. This model is appropriate when the studies in the meta-analysis are diverse in terms of their design, participants, and intervention or exposure. The random-effects model takes into account both within-study and between-study variability, and produces wider confidence intervals to reflect the uncertainty in the estimate of the overall effect size. Based on the different definitions of screen-based disordered eating (and disordered eating in general) in the extant literature, and based also on the wide variability in previous prevalence estimates of SBDE, we assumed that the real effects would fluctuate over time, methodology, and other potentially important variables. Therefore, a traditional random-effects model meta-analysis was carried out using the DerSimonian-Laird method [73].

The assumptions for using random-effects modeling were verified. To account for the variation in effects between studies the logit transformed [PLO] proportions were used in conjunction with the general inverse variance approach [74]. Table 1 presents the prevalence of SBDE, along with 95% confidence interval (95% CI), for each study. The prevalence data were also shown in a forest plot format [75]. In all results the point estimates and corresponding 95% CI referred to the proportion of individuals in the sample who meet or exceed the screening tool cut-off score being used by the authors of the studies.

The *I*^2^ statistic was utilized to assess heterogeneity between studies; a result between 75 and 100% indicates a high level of heterogeneity [76]. We also evaluated heterogeneity using Cochran's Q statistics [77], tau^2^ (τ^2^), and tau (τ) [76]. The *H* statistic [78] is the square root of Cochran’s χ^2^ heterogeneity statistic, divided by the degrees of freedom [76].

Studies whose confidence intervals were outside the confidence interval of the pooled effect were classified as outliers. Because the validity and robustness of a meta-analysis may be compromised by inclusion of outliers, we conducted a sensitivity analysis by replicating the meta-analysis *N* = 66 times, eliminating one different study each time [79].

Funnel plots were created as a basic visual tool to investigate the possibility of publication bias [80]. The trim-and-fill technique [81] was used to create an estimated adjusted point in order to correct for funnel plot asymmetry caused by likely publication bias, although, as explained below, there was no indication of this type of bias.

Meta-analyses for subgroups were conducted to assess further any significantly heterogeneous results (46). As a general rule, a subgroup meta-analysis should be based on three or more studies to ensure that there are sufficient data to support meaningful conclusions [82]. However, in the present meta-analysis analysis it was feasible to use a slightly more conservative cut-off of four or more (i.e., *k* ≥ 4) in order to increase the power of those subgroup analyses.

All findings were represented graphically by forest plots. A meta-regression analysis was conducted to assess amount of variance accounted for by each of the moderator variables [83]. In statistically significant meta-regression models the effect size was reported using *R*^2^, with a minor, medium, or large effect size defined as 1–8%, 9–24%, and 25% of the variation explained, respectively [84].

## Results

### Descriptive

**Fig. 1:**
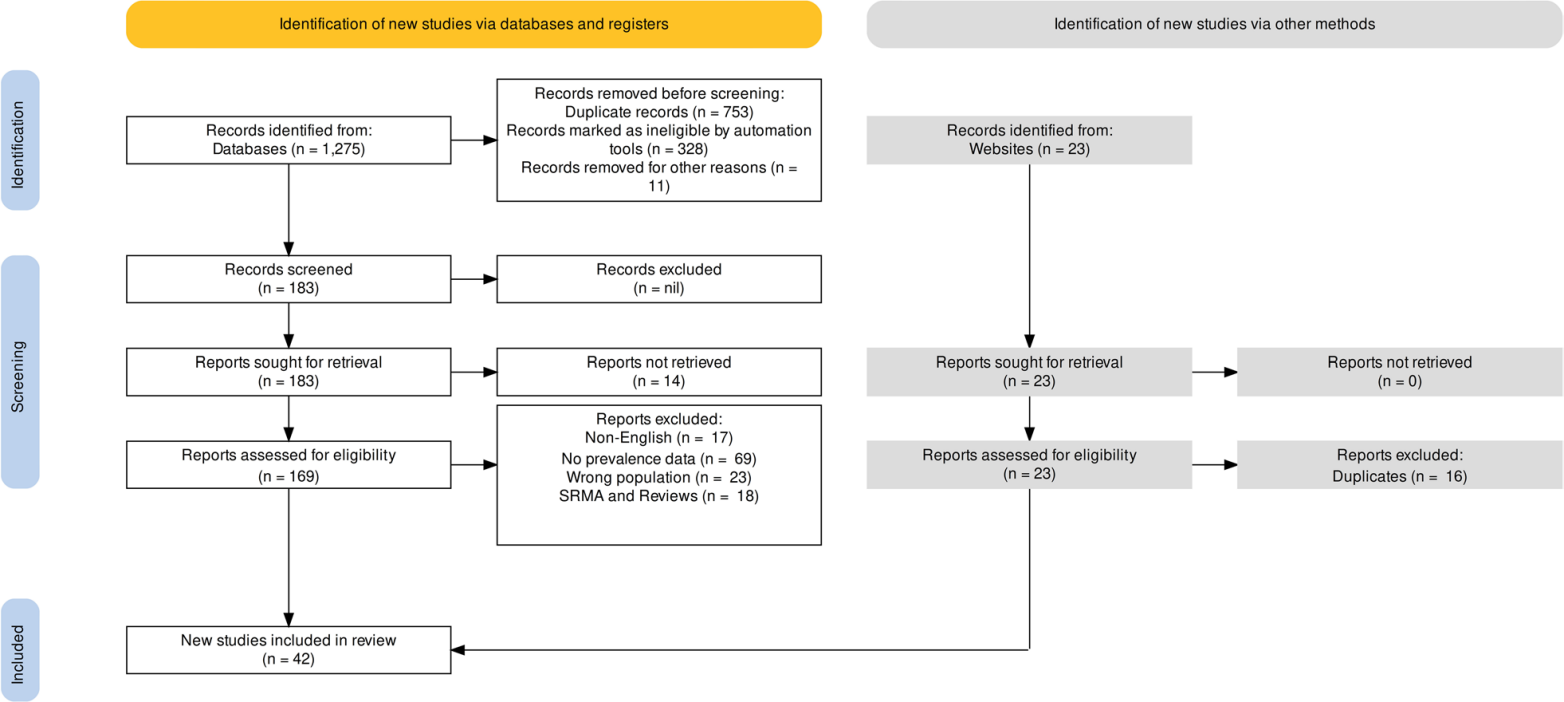
PRISMA 2020 flow diagram for study selection

The literature search, conducted during August and September 2022, yielded 149 studies that eventually produced *K* = 42 studies (66 data points; *N* of participants = 56,282) which met the inclusion and exclusion criteria. Figure 1 show the PRISMA 2020 flow diagram for study selection. The details of the included studies are shown in Table 1. The clear majority (68%) of the studies were cross-sectional, while 29% were longitudinal (cohort design) and 3% used case-control methodology (3%). If the study was longitudinal and began when participants were in high school, then the baseline prevalence was used. If the study used a case-control design, we used the subgroup of “controls”, that is, the healthy screened high-school students that were being compared to the cases with known mental illness.

The EAT-26 was the most commonly used assessment of SBDE, making up 47.5% of the studies (see Table 2). Due to the age criteria set for this review, the mean respondent age was 16 years old [range 14–19 years]. Twenty-five countries were represented in this review (see Table 1), and 80% were categorized as Western culture (21% in Italy). None of the studies meeting the inclusion and exclusion criteria were conducted during the lockdown period of COVID-19 pandemic.

**Table 2:** Random and common effects meta-analysis models of the prevalence of disordered eating in high school students

Analysis	Descriptive	Random-effects meta-analysis	Common-effects meta-analysis	Visual results	Heterogeneity						Moderators			Publication bias	
	K	Pooled results (95% CI)	Pooled results (95% CI)	Forest plot figure no.	H	I^2^ (%)	τ^2^	τ	Q^a^	p	Age	Sex	BMI	Egger's test^b^	Rank test^c^
All Data	66	13.0% [10.0; 16.8]	22.7% [22.3; 23.2]	Figure 4	10.3	99.0	1.5	1.2	6837.3	0.001	0.1	0.5	0.2	NS	NS
By Country				Figure 7						0.001	–	–		–	–
Spain	7	5.7 [3.7; 8.5]	7.3 [6.7; 7.9]		–	96.6	0.3	0.6	177.8						
Italy	14	4.4 [2.2; 8.9]	15.6 [14.7; 16.6]			98.4	1.9	1.4	834.8						
USA	11	21.5 [15.5; 29.0]	19.1 [17.8; 20.4]			94.3	0.4	0.6	174.9						
Australia	4	7.5 [3.0; 17.6]	15.86 [14.64; 17.16]			98.6	0.9	1.0	215.4						
By Culture				Figure 8	–					0.001	–	–		–	–
Western	53	12.1 [8.7; 16.5]	23.8 [23.3; 24.3]			99.2	1.8	1.3	6343.5						
Eastern	13	17.0 [12.6; 22.7]	16.8 [15.9; 17.8]			96.6	0.4	0.6	349.0						
By COVID-19				Not Shown	–					0.001	–	–		–	–
Yes	0	–	–			–	–	–	–						
No	66	13.0 [10.0; 16.8]	22.7 [22.3; 23.2]			99.0	1.5	1.2	6837.3						
By Tool				Figure 9	–					0.001	–	–		–	–
EAT26	15	14.7 [10.6; 20.0]	15.1 [14.4; 15.9]			96.4	0.5	0.7	388.8						
DAWBA	4	29.3 [22.2; 37.4]	30.8 [29.5; 32.1]			98.3	0.1	0.4	177.3						
EAT40	6	8.2 [4.3; 14.8]	10.7 [9.9; 11.5]			97.6	0.7	0.8	206.7						
EDI2	4	38.8 [27.1; 52.0]	47.9 [46.2; 49.5]			98.6	0.3	0.5	213.7						
EAT	4	3.3 [1.0; 11.1]	9.1 [8.0; 10.4]			96.0	1.6	1.3	75.7						
BITE	6	2.7 [1.3; 5.6]	4.8 [4.1; 5.5]			95.5	0.8	0.9	110.4						
EDEQ	6	28.8 [21.0; 38.2]	25.2 [23.6; 26.8]			91.3	0.3	0.5	57.8						
By Design				Not Shown	–					0.001	–	–		–	–
Cross-sectional	45	13.6 [10.0; 18.4]	23.6 [23.1; 24.2]			99.1	1.4	1.2	4781.0						
Cohort	19	10.4 [6.1; 17.0]	19.7 [19.0; 20.4]			99.0	1.6	1.3	1861.8						
Case-control	2	35.5 [20.8; 53.5]	34.1 [31.1; 37.3]			98.2	0.3	0.5	56.4						
By time framework				Figure 10	–					0.001	–	–		–	–
1990–1994	3	3.3 [2.54; 4.4]	3.3 [2.5; 4.4]			0.0	0	0	1.6						
1995–1999	7	11.8 [6.7; 20.0]	11.02 [10.30; 11.8]			98.1	0.7	0.8	315.1						
2000–2004	11	13.7 [7.2; 24.6]	25.2 [24.2; 26.3]			99.2	1.5	1.2	1281.5						
2005–2009	26	8.9 [5.3; 14.5]	15.1 [14.4; 15.7]			98.8	2.0	1.4	2107.8						
2010–2014	6	24.9 [13.7; 40.8]	22.0 [20.6; 23.5]			97.1	0.8	0.9	173.2						
2015–2019	8	27.4 [18.3; 38.9]	41.02 [39.8; 42.2]			98.7	0.6	0.7	533.5						
2020–2024	5	21.7 [14.3; 31.7]	24.0 [22.8; 25.2]			96.9	0.3	0.6	130.5						

K included studies numbers,

I^2^ Statistic refereed to the percentage of variation across samples due to heterogeneity rather than chance

τ^2^ Describe the extent of variation among the effects observed in different samples (between-sample variance)

H Describe confidence intervals of heterogeneity

^a^Significant differences between samples in meta-analysis

^b^Detects publication bias in meta-analysis

^c^Represent the correlation between effect sizes and sample variation

### Quality assessment

**Fig. 2:**
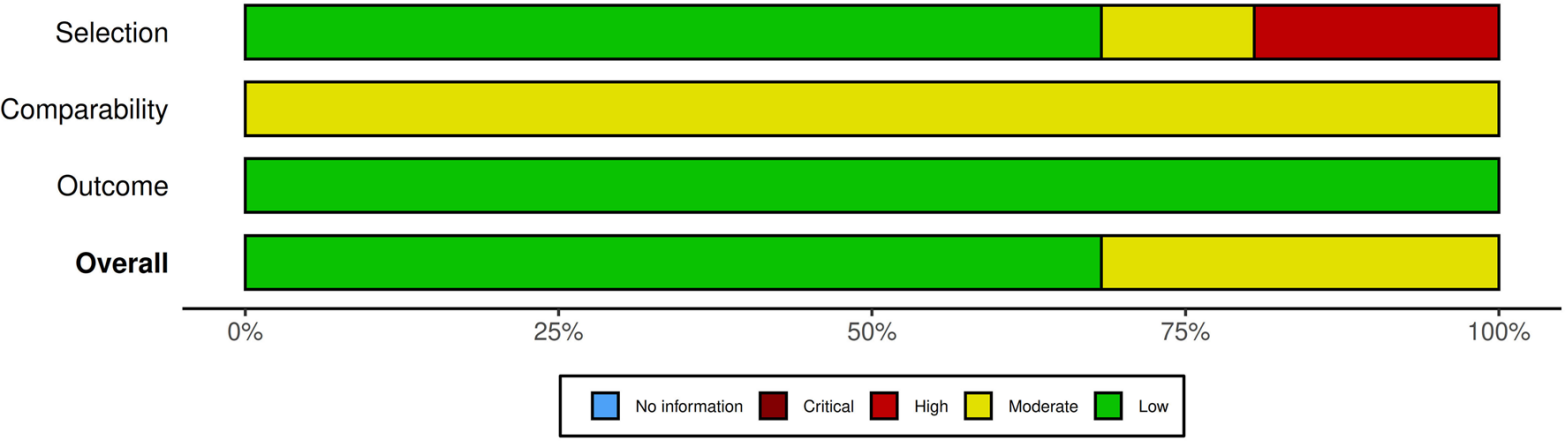
Summary plot of the assessment of the risk of bias

**Fig. 3:**
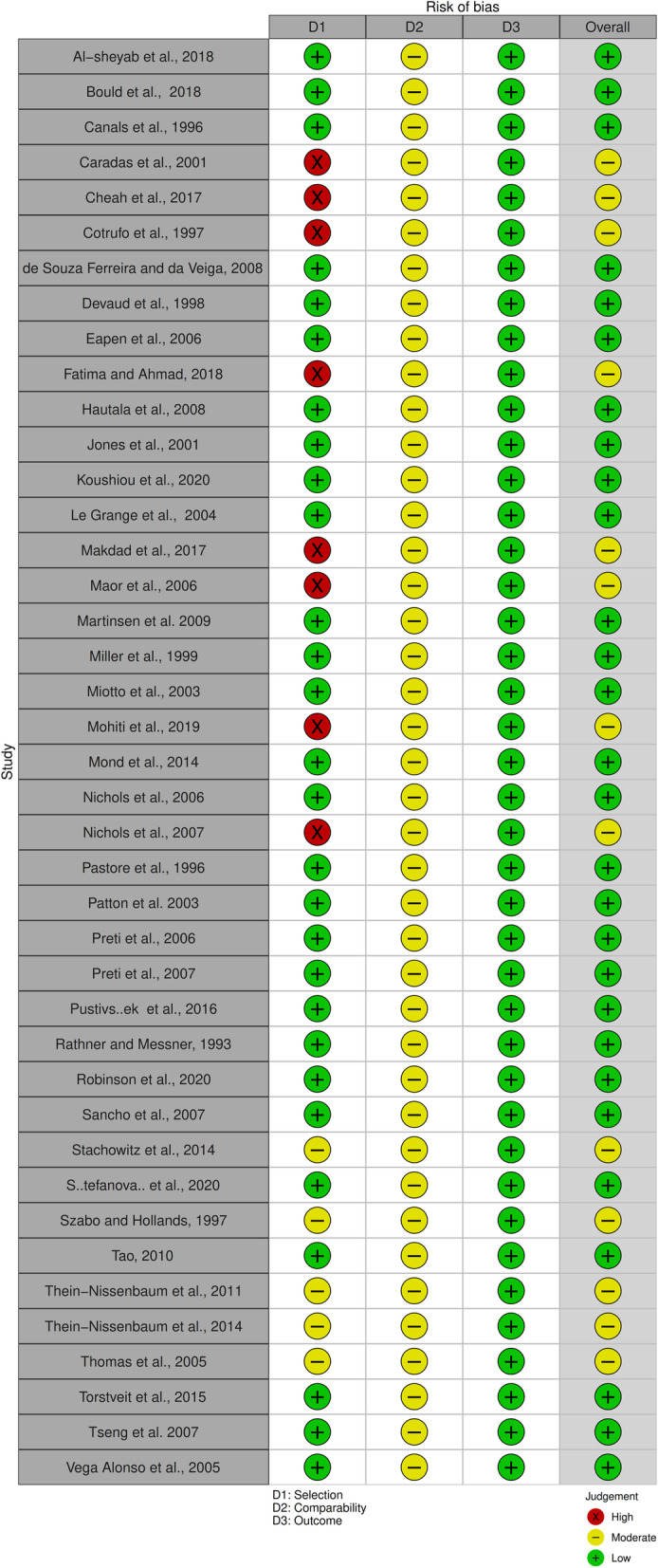
Traffic light plot of the assessment of the risk of bias

Figure 2 shows summary plot of the assessment of the risk of bias. Figure 3 shows the traffic light plot that summarizes all risk of bias assessments in each domain for each of the studies, along with the overall risk was used. Sixty-eight percent of the studies had a low overall risk of bias, while for the remaining 32% the overall risk was moderate.

### Meta-analysis of the overall prevalence of screen-based disordered eating

**Fig. 4:**
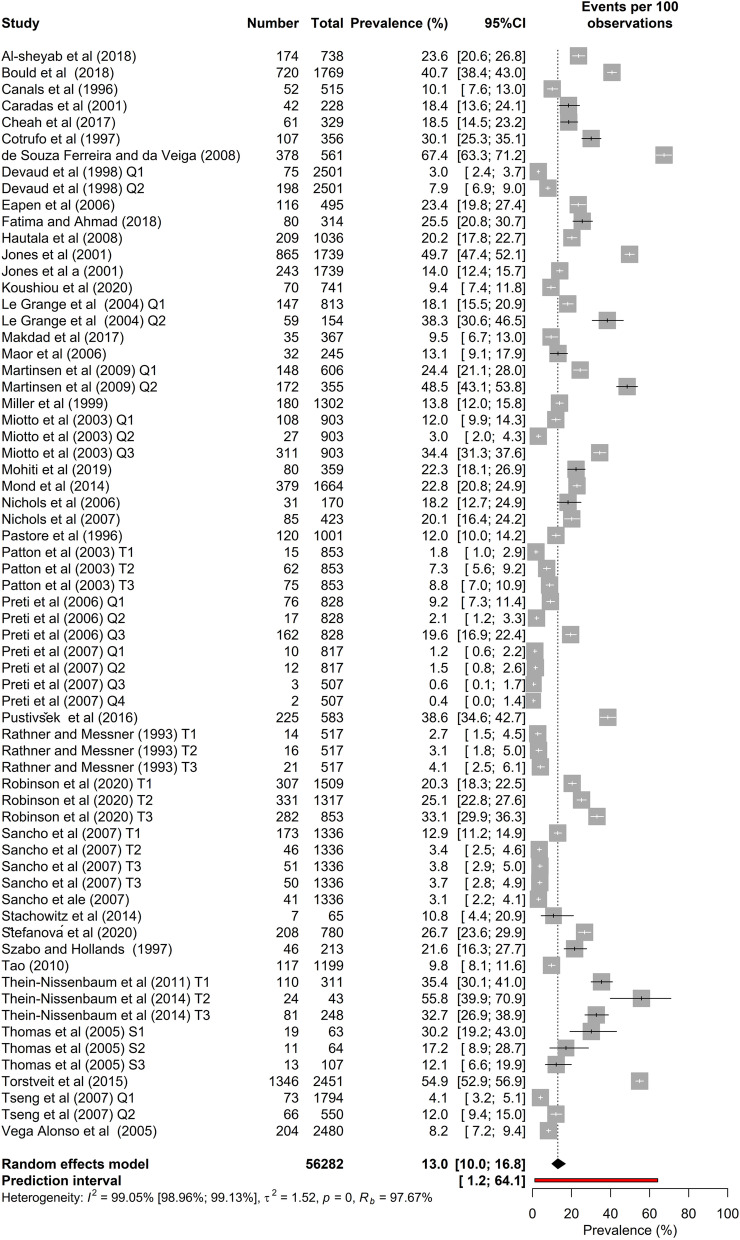
Classical meta-analysis of disordered eating in high school students

The raw prevalence data and meta-analysis results are presented in Fig. 4. According to the random-effects meta-analysis, the SBDE prevalence among high school students (*K* = 66, *N* = 56,282) was [95% CI] = 13.0% [10.0–16.8]. As expected, there was high and statistically significant heterogeneity in the prevalence estimates, *I*^2^ = 99.0% [99.0–99.1], *τ* [95% CI] = 1.23 [1.1; 1.5], τ2 [95% CI] = 1.51 [1.1; 2.4], *H* [95% CI] = 10.26 [9.8–10.7], *p*-value of Cochran’s *Q* = 0.001.

**Fig. 5:**
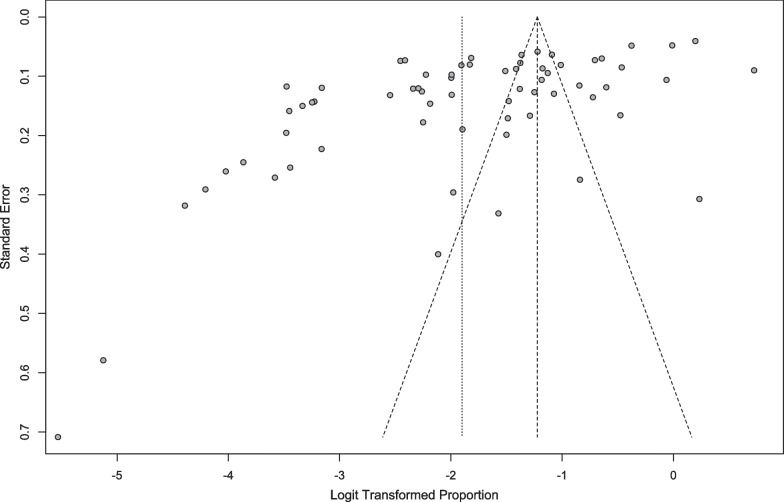
Funnel plot of disordered eating in high school students

**Fig. 6:**
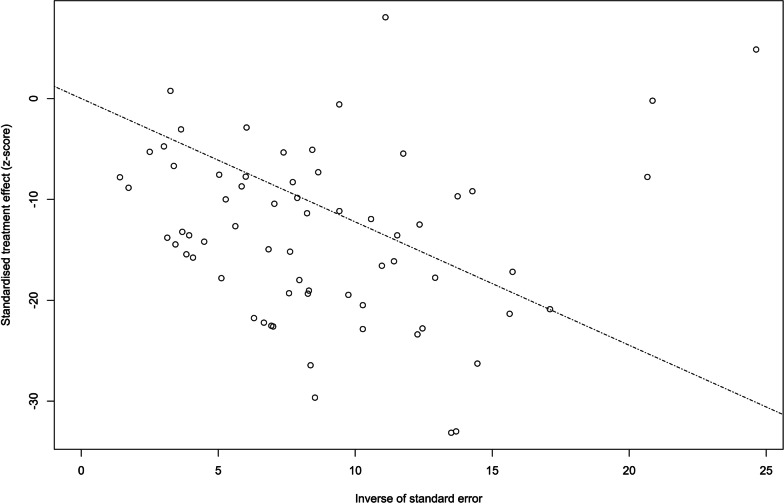
Radial plot of disordered eating in high school students

Visual inspection of the funnel (Fig. 5) and radial plots (Fig. 6), as well as non-significant Egger's regression and Peter's tests (*p* > 0.05), indicated that our data are free of publication bias.

### Confounder (moderator) analysis

Table 2 presents the analyses of subgroups, with the stipulation that in each category *k* ≥ 4.

### Age, BMI, and gender

Meta-regression analyses demonstrated that neither age, BMI, nor percentage of females in the sample was a statistically significant moderator (confounder) of prevalence effect size, *p* = 0.1, *p* = 0.2 and *p* = 0.5, respectively. The effect sizes were very small: age *R*^2^ =  ~ 3.0%; BMI *R*^2^ =  ~ 3.3%; and gender *R*^2^ =  < 1%. Due to lack of significance in all three variables, no interaction was tested.

### Country and culture

We examined the weighted prevalence levels as a function of the country in which the data were collected. These varied tremendously, and, as noted above, for many countries the number of studies meeting the inclusion and exclusion criteria was very low. Brazil (*k* = 1, *N* = 378) at 67.4% [63.3–71.2] had the greatest prevalence of SBDE among high school students, followed by Norway (*k* = 3, *N* = 3,412) at 41.9% [27.3–58]) and the United Kingdom (*k* = 1, *N* = 1,769) at 40.7% [38.4–43.0]. Italy, which had the highest number of studies of SBDE among high school students (*k* = 14, *N* = 9,748), had the lowest prevalence at 4.4% [2.1–8.9], followed by Switzerland (*k* = 2, *N* = 5,002) at 4.9% [2.5–9.5].

**Fig. 7:**
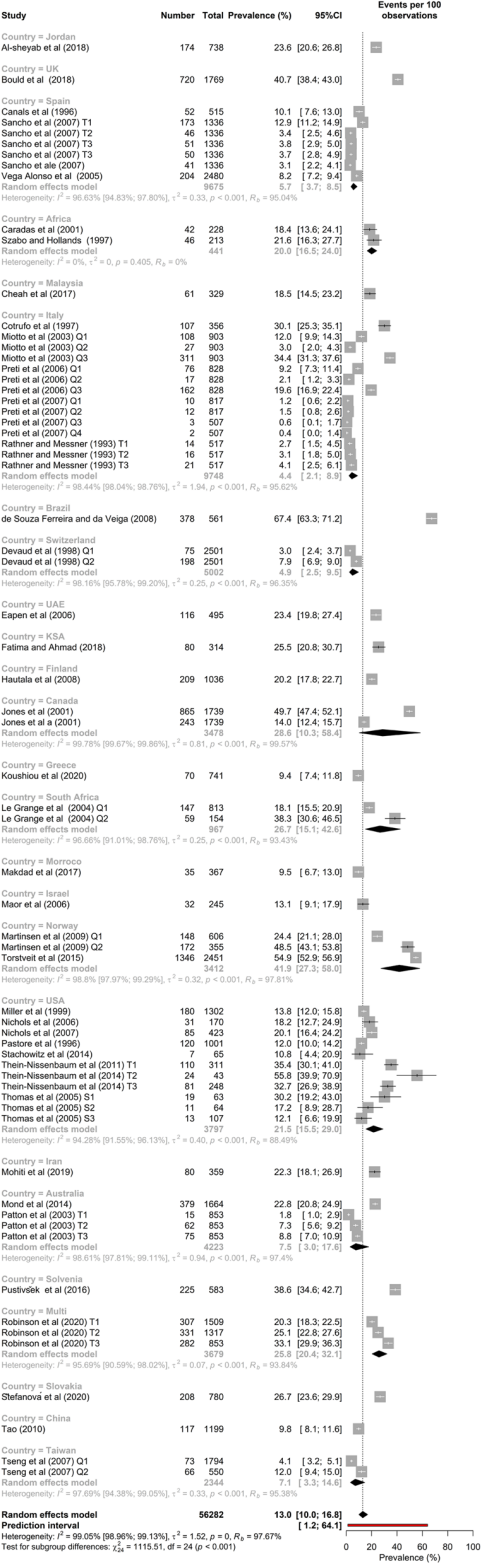
Subgroup meta-analysis by Country

Figure 7 shows the subgroup meta-analysis of SBDE in high school students by country. There were four countries in which four or more studies have been conducted and published. For these countries a clear pattern is evident; the prevalence of SBDE in the USA was ~ 21.5%, while in the others (all Western countries) the prevalence range was ~ 4.5% through 7.5%. The subgroup heterogeneity meta-analysis for those four studies showed that this difference was statistically significant, *p* < 0.001.

**Fig. 8:**
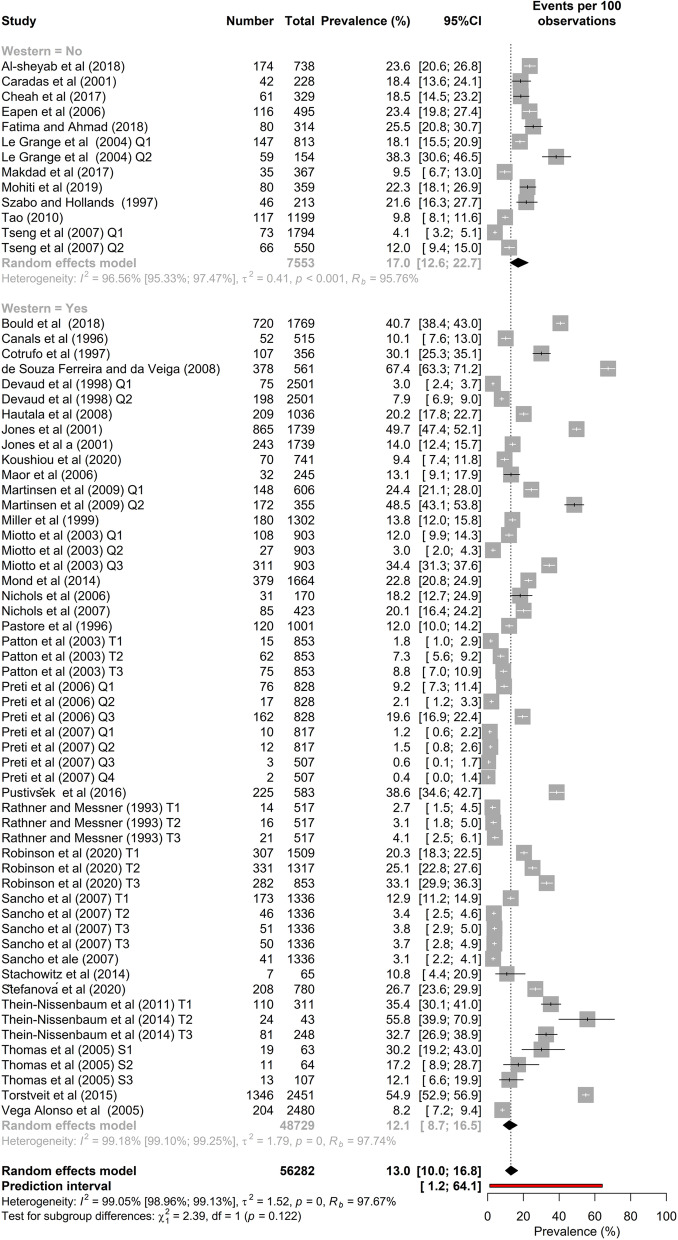
Subgroup meta-analysis by Culture

As a group, Western countries (*k* = 53, *N* = 48,729) have a slightly lower prevalence of SBDE (12.1%; 95% CI = 8.7–16.5) among high school students than do non-Western countries (*k* = 13, *N* = 7553) at 17.0% (12.6–22.7), but that difference was not statistically significant (*p* = 0.12). Figure 8 shows subgroup meta-analysis of disordered eating in high school students by culture.

### Measures of SBDE

**Fig. 9:**
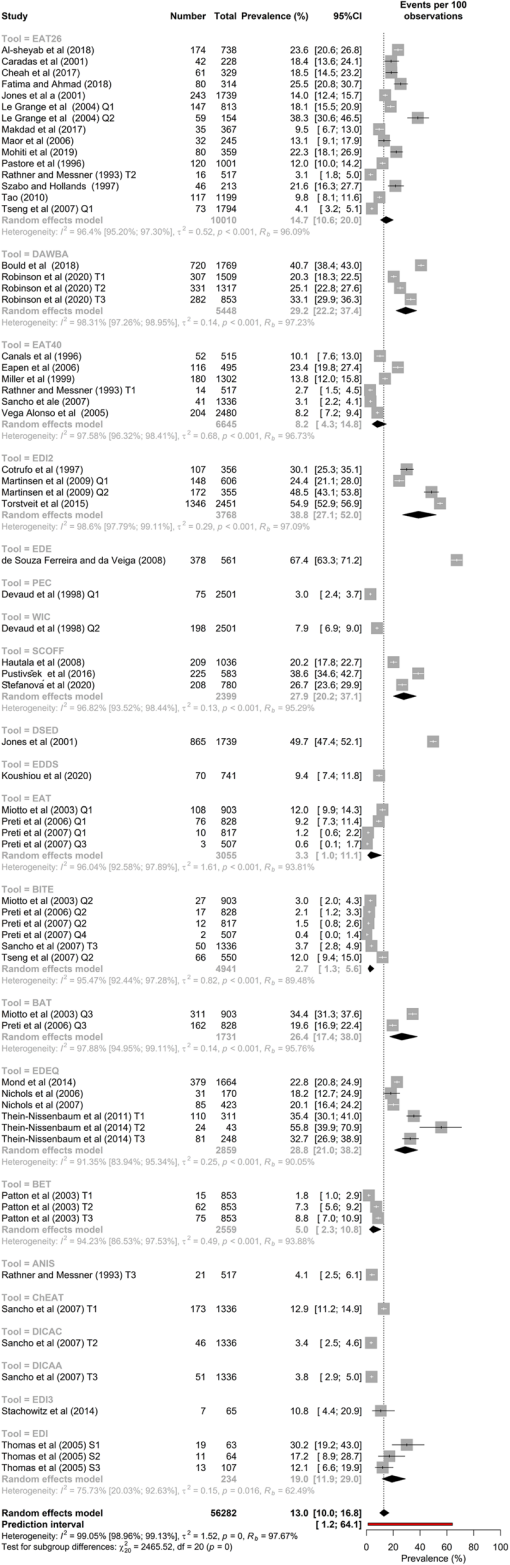
Subgroup meta-analysis by disordered eating measure

The most frequently used measures were the EAT-26, which yielded a prevalence of 14.7% ([10.6–20.0], *k* = 15, *N* = 10,010), followed by the BITE (*k* = 6, *N* = 4,941) = 2.7% [1.3–5.6]) and the EDE-Q (*k* = 6, *N* = 3,172) = 28.8% [21.0–38.2]. Considering all 21 measures, the EDE-Q (*k* = 1, *N* = 561) produced the highest prevalence at 67.4% [63.3–71.2], while the BITE (*k* = 6, *N* = 4,941) yielded the lowest prevalence at 2.7% [1.3–5.6]. Meta-analysis showed that, as expected, amongst the many different measurement tools used in the studies (Table 2), there was significant heterogeneity, *I*^2^ = 99%, *τ*^2^ = 1.52, *p* = 0.001. Of the measures used in four or more studies, the EDI-2 yielded the highest prevalence at 38.8% ([27.1–52.0], *k* = 4, *N* = 3,768). Figure 9 shows subgroup meta-analysis of disordered eating in high school students by measure.

### Time framework

**Fig. 10:**
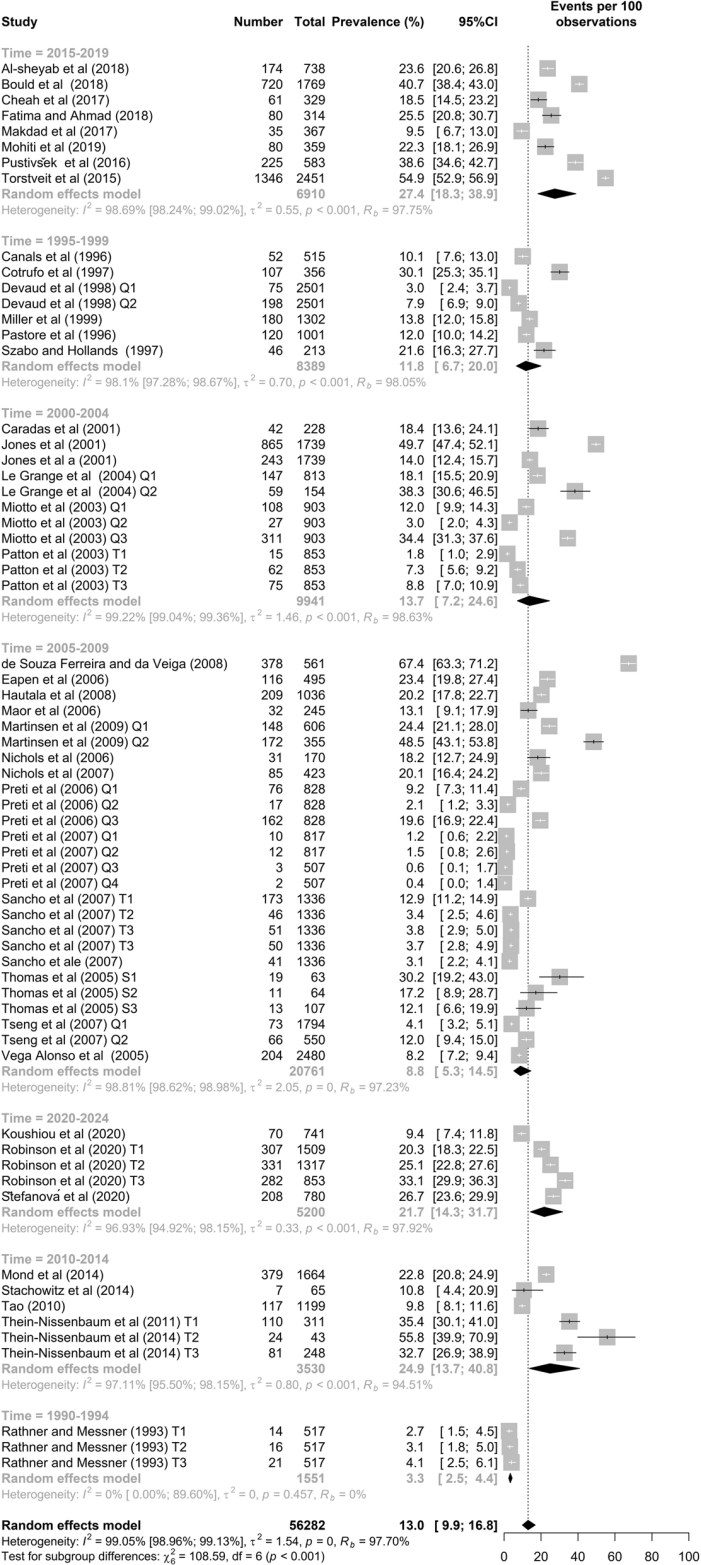
Subgroup meta-analysis by timeframe/year

In regard to the date of publication for the studies included in this meta-analysis, the period between 2015 and 2019 (*k* = 8) yielded the highest prevalence of SBDE (27.4%; 18.3–38.9), whilst the lowest prevalence (3.3%; 2.54–4.4; *k* = 3) was in the period between 1990 and 1994. The prevalence In the other periods of time ranged between 11.8% through 24.9%. Figure 10 shows subgroup meta-analysis of disordered eating in high school students by time framework of data collection.

## Discussion

The purpose of this meta-analysis was to estimate the prevalence of disordered eating in high school students, based on published studies using screening measures validated with adults. The search and selection process resulted in 42 studies, conducted in 25 countries, that contributed 66 data points (total *N* = 56,282). This meta-analysis produced an estimate of 13% as the global prevalence of SBDE in high school students.

Given the physical and mental health consequences of disordered eating, and given its status as a risk factor for, or prodromal feature of, clinically significant eating disorders, the figure of 1 in 8 high school students with SBDE stands out as a problem in need of attention from public health officials, psychologists, psychiatrists, pediatricians, parents, and educators. Other than some notable successes with older high school students, *universal* prevention efforts with adolescents ages 14 through 17 have in general had very limited success [11]. Two prevention programs in the selective-indicated range of the Mental Health Intervention Spectrum [11], the dissonance-based Body Project and the Healthy Weight Program, have been effective over several years of follow-up in reducing risk factors and preventing eating disorder onset in at-risk female high school students [84–86]. However, there are yet no effective programs for at-risk adolescents in high school who identify as boys or nonbinary. One recent attempt to apply significant components of a Body Project program that showed some promise with undergraduate males [87] did produce any significant effects for males in grades 9 and 10 [88]. Thus, overall, there is a great need for work all along the spectrum of health promotion and universal prevention. This research should be incorporated with mental health literacy programming designed to teach adolescents and adults who care about them (e.g., parents, teachers, coaches, clergy, physicians, dentists) to identify disordered eating and support adolescents in getting help for this set of problems.

The prevalence estimate of 13% was, contrary to expectations, not significantly moderated by age, proportion of females in the sample, the sample’s mean BMI level, or whether the country in which the data were collected was Western or non-Western. In fact, contrary to what we expected, the prevalence of SBDE was higher in non-Western countries. Interestingly, the prevalence of SBDE in high school students in Middle East Arabic countries was 24% (23.6–25.5; *k* = 4), confirming that there are multiple sociocultural pathways to DE in populations, and supporting the contention that Westernization is a construct of very limited usefulness (6).

The lack of a correlation between SBDE and age is likely due in part to the truncated age range, and the type of data typically provided made it impossible to compare younger versus older high school students. This comparison is likely an important one for future research because the modal age range for ED onset is late adolescence and emerging adulthood. Thus, as expected, the figure of 13% for SBDE—in the context of its 95% confidence interval (10–17%)—is substantially less than the estimated prevalence of 19.7% ([17.9–21.6], *K* = 105 data points; total N = 149,629) of SBDE in university and college undergraduates from 40 countries (7). If those two estimates prove to be robust, then it would be expected that the prevalence of SBDE would be greater in adolescents in 17 and 18 (~ 17%) than in those 15 and 16 (~ 9–10%). One possibility is that this expected linear pattern applies only to students, as evidence from a longitudinal study in the USA indicates that in general disordered eating behaviors either stabilize or decrease from adolescence into young adulthood (28). However, another possibility with important implications for prevention emerges from a recent longitudinal study conducted in Canada [89], which identified a distinct trajectory, present in slightly less than one third of youth, defined by a sharp increase in disordered eating between ages 12 and 15, leading to high and steady levels between ages 15 and 20. By contrast, nearly 70% of the youth in this study had consistently low levels of disordered eating across adolescence.

The lack of a correlation between prevalence effect size and either BMI or gender is surprising, because a lot of research with adolescents supports the relevance of these variables for body image, disordered eating, and eating disorders. Moreover, in our recent meta-analysis of moderators of SBDE in college and university students (7) we found that BMI had a large positive effect, while being female had a small positive effect. In the samples of high school students that we located for the present meta-analysis the range of mean BMI was only 18–23, and, more importantly, 30 of the 42 (71.4%) studies had a mean BMI of 21. Thus, it is likely that this lack of variability in mean BMI produced a negligible correlation with the prevalence of SBDE. In the studies comprising our meta-analysis of university students the range of mean BMI was greater (~ 17 to ~ 26) and 68 of 89 studies (76.4%) reported a mean BMI in their sample of > 22. In future studies of SBDE it is worth investigating whether the significant *increase* in weight from late adolescence (age 18, the end of high school) to emerging adulthood in the USA [90] is taking place in other countries; and if so, whether this weight gain is a key variable in producing significant increases in weight and shape concerns and related disordered eating in the context of university life and its increased expectations for identity development, autonomy in general, self-control over food intake, sexual attractiveness and sexual behavior, and academic success.

The lack of a gender difference in the prevalence of SBDE in high school students in 25 countries merits further research. In a longitudinal analysis of data from several cohorts of U.S. participants in Project EAT, Simone et al. [64] found that in adolescence and late adolescence/emerging adulthood females were clearly more likely than males to report engaging in unhealthy weight control behaviors, and they were much more likely to report binge eating. Similarly, in their longitudinal study Ferreiro et al. [63] found no difference in disordered eating between females and males at mean age ~ 11 years, but higher scores among females at age ~ 13, and at age ~ 15 as well, if they also had higher levels of depressive symptoms.

To advance our understanding of SBDE in high school in ways that facilitate prevention, it will be important to not only survey self-identified males, females, and non-binary individuals, but also to examine the temporal [89] and symptom patterns of disordered eating in more detail. In a recent study of 729 Taiwanese adolescents ages 13 through 16, Chen et al. [91] found an overall prevalence of SBDE of 11.4%, which is very similar to the mean estimate of 13.0% in our meta-analysis. There was very little difference between females (11.6%) and males (11.2%) in the prevalence of SBDE based on the EAT-26, but there were significant differences on individual items of that questionnaire. Boys were more likely to want to have their stomachs feel empty and to have the urge to vomit after eating. Girls were much more likely to report cognitive features: fear of being overweight, preoccupation with body fat, and preoccupation with becoming thinner.

More studies, using established epidemiological methods (e.g., representative sampling), are needed in order to clarify the prevalence and correlates of SBDE in high school students. In this regard the published research to date has been limited to samples from only 25 countries, that is, about 10% of the approximately 250 independent nation states, dependencies, territories, and other entities recognized by both the United Nations and the USA. Notably absent in the literature are studies (meeting our criteria) from Latin American countries (other than one from Brazil), and there were either no studies or only one from several countries who have contributed significantly to the literatures on eating disorders and/or prevention, such as the United Kingdom, Sweden, France, Germany, and Canada.

Researchers seeking to understand the prevalence of disordered eating as a multifaceted construct have many screening tools from which to choose (Table 1). As discussed below in the limitations section, there is a need for basic research to establish the validity of any of the possible screening tools in relation to their sensitivity and specificity in predicting any full-blown eating disorder or specific eating disorders. Pending that challenging work, based on the substantial differences in prevalence estimates across the studies included in our meta-analysis, and in order to facilitate comparisons across studies from different countries while avoiding estimates that are almost certainly far too high or too low, we recommend use of the EAT-26 [92] plus one other instrument such as the Eating Disorder Diagnostic Scale (EDDS; [93]). The EDDS, which was used in only one study (70) included in this meta-analysis, is a valid and widely used measure of ED symptoms, and therefore it can add behavioral information to the screening items on the EAT-26. If the EAT-26 is impractical due to its length, we recommend substituting the 5-item SCOFF, which, according to Table 1, has been used in only three studies of SBDE in high school students [33, 50, 55]. This would facilitate comparison with data from college and university students; in our recent meta-analysis we found 25 studies that administered the SCOFF to samples of these students.

### Study strength and limitations

To the best of our knowledge, this is the first meta-analysis of the prevalence of screen-based disordered eating among high school students. Thus, the findings of this meta-analysis are unique and do not overlap with previous meta-analyses on this topic that were exclusively focused on university students or medical students. The large number of studies (*N* = 42) and participants included (*N* = 55,282 participants) strengthens this statistical review.

A major limitation is that, as confirmed by two recent reviews [94, 95] (BB, CC), there currently is a very pronounced lack of studies of the accuracy of screening instruments for determining the “at risk for eating disorders” status of adolescents in the high school age range and in general. If public health efforts to acknowledge and understanding more fully screen-based disordered eating, and to thereby fashion prevention policies and program, are to proceed, this problem must be addressed.

Other limitations include the reliance of many studies on convenience rather than representative samples, and our limiting of articles reviewed to those published in English and Arabic. The limited nature of the information in the Participants sections, and thus in the data analysis of many studies, also ruled out statistical examination of potentially important moderating variables, such as sexual orientation, ethnicity, and immigration status. The absence of information about the latter two variables means that the distinction between Western and non-Western samples was based on country of residence, which does not necessarily equate to culture. Thus, we caution against conflating the two and suggest that future research consider more nuanced approaches to cultural classification.

## Conclusion

The prevalence of screen-based disordered eating in a very large sample of high school students from 25 countries appears to be 13%. Although this is considerably less than the estimate of 20% yielded by our parallel meta-analysis of college and university students in 42 countries, 4 in every 30 high students with multiple indicators of disordered eating attitudes and behaviors merits the attention of mental health professionals, public health officials, educators, and parents. Disordered eating is a problem in its own right, as well as a risk factor for eating disorders, and our meta-analysis strongly suggests that in high school it affects females and males to an equal degree. Consequently, it is long past time for multiple projects to development and evaluation of health promotion efforts, prevention programs, and mental health literacy for high school students.

A figure of 13% also merits further research to refine this estimate by (a) conducting basic research on the accuracy of eating disorder screening measurements in samples ages 14 through 17; (b) examining representative samples in more countries in general and Latin American countries in particular; (c) clarifying the relationships between SBDE and age throughout the different phases of adolescence and emerging adulthood; and (d) using multivariate statistics to determine whether there are meaningful forms of disordered eating and whether these are associated with variables such as gender and BMI.

## Data Availability

Data is available upon a valid and reasonable request from the corresponding author (H.J.).
